# A cross-sectional study of *Leishmania infantum* infection in stray cats in the city of Zaragoza (Spain) using serology and PCR

**DOI:** 10.1186/s13071-021-04682-w

**Published:** 2021-03-25

**Authors:** Maria Magdalena Alcover, Asier Basurco, Antonio Fernandez, Cristina Riera, Roser Fisa, Ana Gonzalez, Maite Verde, Ana María Garrido, Héctor Ruíz, Andrés Yzuel, Sergio Villanueva-Saz

**Affiliations:** 1 Departament de Biologia, Salut I Medi Ambient, Facultat de Farmacia, Universitat de Barcelona, Barcelona, Spain; 2 Laboratorio de Inmunopatología Clínica, Facultad de Veterinaria, Universidad de Zaragoza, Zaragoza, Spain; 3 Departamento de Patología Animal, Facultad de Veterinaria, Universidad de Zaragoza, Zaragoza, Spain; 4 Departamento de Farmacología Y Fisiología, Facultad de Veterinaria, Universidad de Zaragoza, 50013 Zaragoza, Spain

**Keywords:** FeLV, FIV, *Leishmania infantum*, Serology, PCR, Blood, Cat

## Abstract

**Background:**

Feline leishmaniosis is a vector-borne parasitic disease caused by *Leishmania* spp. *Leishmania* infection in dogs is prevalent in the Mediterranean basin, but in other animals, such as cats, it could also play a role in the epidemiology of the disease. Information on the geographical distribution and epidemiological features of *L. infantum* infection in cats is scarce, particularly in urban stray cats living in regions where canine leishmaniosis is endemic. As diagnosis can be challenging, combining different serological and molecular methods is a useful approach. Our aim was to investigate the prevalence of infection of *L. infantum* in apparently healthy stray cats in an endemic region of Spain (Zaragoza city) using serological and molecular methods, and to compare the results of the different techniques.

**Methods:**

The prevalence of *Leishmania* infection was studied in stray cats captured in urban and peri-urban areas of Zaragoza. Blood was collected from each animal for serology and molecular analysis. Three serological methods, namely the immunofluorescent antibody test (IFAT), enzyme-linked immunosorbent assay (ELISA) and western blot (WB), were used to detect *L. infantum* antibodies and a real-time PCR (qPCR) assay was used to detect *L. infantum* DNA. The results were analyzed by Fisher’s exact test and Cohen’s kappa statistic (*κ*)  to assess the level of agreement between the diagnostic techniques.

**Results:**

Serological analysis of blood samples from 180 stray cats revealed 2.2% (4/179) *Leishmania* infection positivity by IFAT, 2.8% (5/179) by ELISA and 14.5% (26/179) by WB. *Leishmania* DNA was detected by qPCR in 5.6% (10/179) of the cats. Sixteen cats (8.9%) tested positive by only one serological technique and four tested positive by all three serological methods used. The overall rate of infected cats (calculated as the number of cats seropositive and/or qPCR positive) was 15.6%, and only two cats tested positive by all the diagnostic methods. A significant association was found between male cats and a positive qPCR result. Comparison of the techniques revealed a fair agreement in seropositivity between blood qPCR and IFAT (*κ* = 0.26), blood qPCR and ELISA (*κ* = 0.24), WB and ELISA (*κ* = 0.37) and WB and IFAT (*κ* = 0.40). The highest agreement between seropositive results was between IFAT and ELISA (*κ* = 0.89), and the lowest was between blood qPCR and WB (*κ* = 0.19). The prevalence of the feline leukemia virus antigen was 4.49% (8/178 cats) and that of the feline immunodeficiency virus (FIV) antibody was 6.74% (12/178), while co-infection with both retroviruses was observed in one female cat (1/178). *Leishmania* ELISA and IFAT seropositivity were statistically associated with FIV status by the chi-square test.

**Conclusions:**

The results obtained in this study, using serological tests and qPCR, indicate the existence of *L. infantum* asymptomatic infection in apparently healthy stray cats in the city of Zaragoza, an endemic area in Spain.

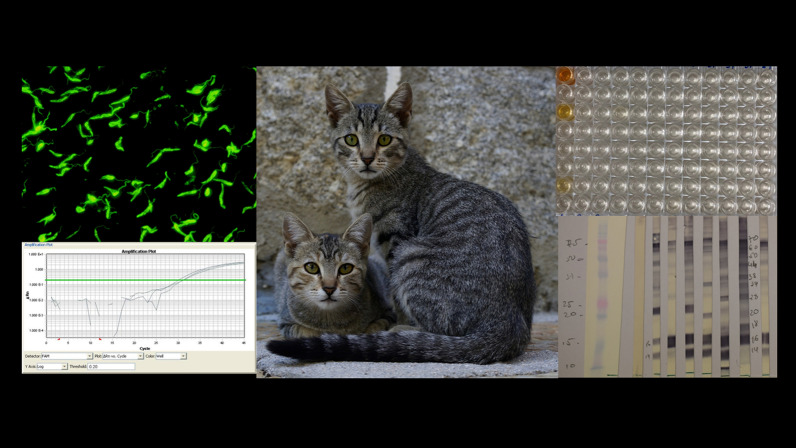

## Background

Leishmaniosis is a complex vector-borne disease caused by different species of the genus *Leishmania* that is endemic in 88 countries of southern Europe, Africa, Asia and South and Central America. More than 350 million people are estimated to be at risk of the disease [1], with dogs being the main reservoir for *Leishmania infantum* infection. Based on seroprevalence studies and environmental variables, 23.2% of dogs in Italy, Spain, France and Portugal are estimated to be infected [2]. Although *Leishmania* infection in dogs is well documented, there is a need for more information on its prevalence in cats in the same areas.

Various phlebotomine species are implicated in the transmission of *L. infantum* in Europe, of which only two are found in Spain: *Phlebotomus ariasi* and *Phlebotomus perniciosus* [3]. Female *Phlebotomus* spp. feed on a variety of vertebrate reservoirs, including humans, livestock, dogs, wild rabbits, hares, rodents and cats [4], with variable impacts on the epidemiology of leishmaniosis. Colonies of stray cats could play a role in the maintenance of the *Leishmania* life-cycle in an urban environment, where cats are naturally exposed to active vectors.

Free-roaming cats in European cities are a potential source of zoonotic diseases. In the case of *Leishmania*, cats can be infected for several years without exhibiting clinical signs [5]. In dogs, clinical manifestations of infection may range from absent or mild to severe and even fatal disease. This variability is thought to be the result of a cell-mediated immune response from the host, which may be influenced by the genetic background. A similar pattern of humoral and cell-mediated adaptive immune response is detected in cats from endemic areas of *L. infantum* [6]. The most common clinical signs of feline leishmaniosis (FeL) include peripheral lymphadenomegaly, cutaneous and mucocutaneous lesions (e.g. nodular and/or ulcerative dermatitis), generalized weakness, weight loss, anorexia and ocular and oral lesions. Some of the most frequent clinicopathological abnormalities seen in FeL are non-regenerative anemia, hyperproteinemia with hyperglobulinemia and hypoalbuminemia and proteinuria [7].

To carry out the complex diagnosis of FeL, serological and molecular techniques are commonly employed in clinical and epidemiological studies [8]. Serological studies have included the immunofluorescent antibody test (IFAT) [9], the direct agglutination test (DAT) [10], the enzyme-linked immunosorbent assay (ELISA) [11] and western blot (WB) [12]. In contrast with serology techniques, molecular diagnostic techniques are not restricted to bodily fluids, but can also be applied to bone marrow, the lymph node, spleen and skin tissues. Nevertheless, blood is the most commonly used sample for molecular testing in epidemiological surveys.

In an epidemiological survey carried out in the area of Barcelona (Spain), molecular test results indicated that *L. infantum* subclinical infections outnumber clinical infections in cat populations in endemic regions [13]. As serological methods may not be sufficiently sensitive to bring subclinical infections to light, a combination of at least two positive results by molecular and serological techniques is recommended for a more accurate estimation of *Leishmania* infection [14]. Several studies have provided seroprevalence and molecular data in endemic southern European countries, particularly Spain [15], France [16], Portugal, [17] and Italy [18], but considerable gaps in regional data remain to be filled. Given the absence of epidemiological studies on feline infection in the city of Zaragoza, an endemic region of Spain, the aims of the present study were: (1) to investigate the prevalence of *L. infantum* infection in stray cats in Zaragoza using serology and quantitative (qPCR) assays; and (2) to evaluate the screening results of apparently healthy stray cats living in an endemic region by comparing serological and qPCR test data.

## Methods

### Study areas, cats and sampling

**Fig. 1:**
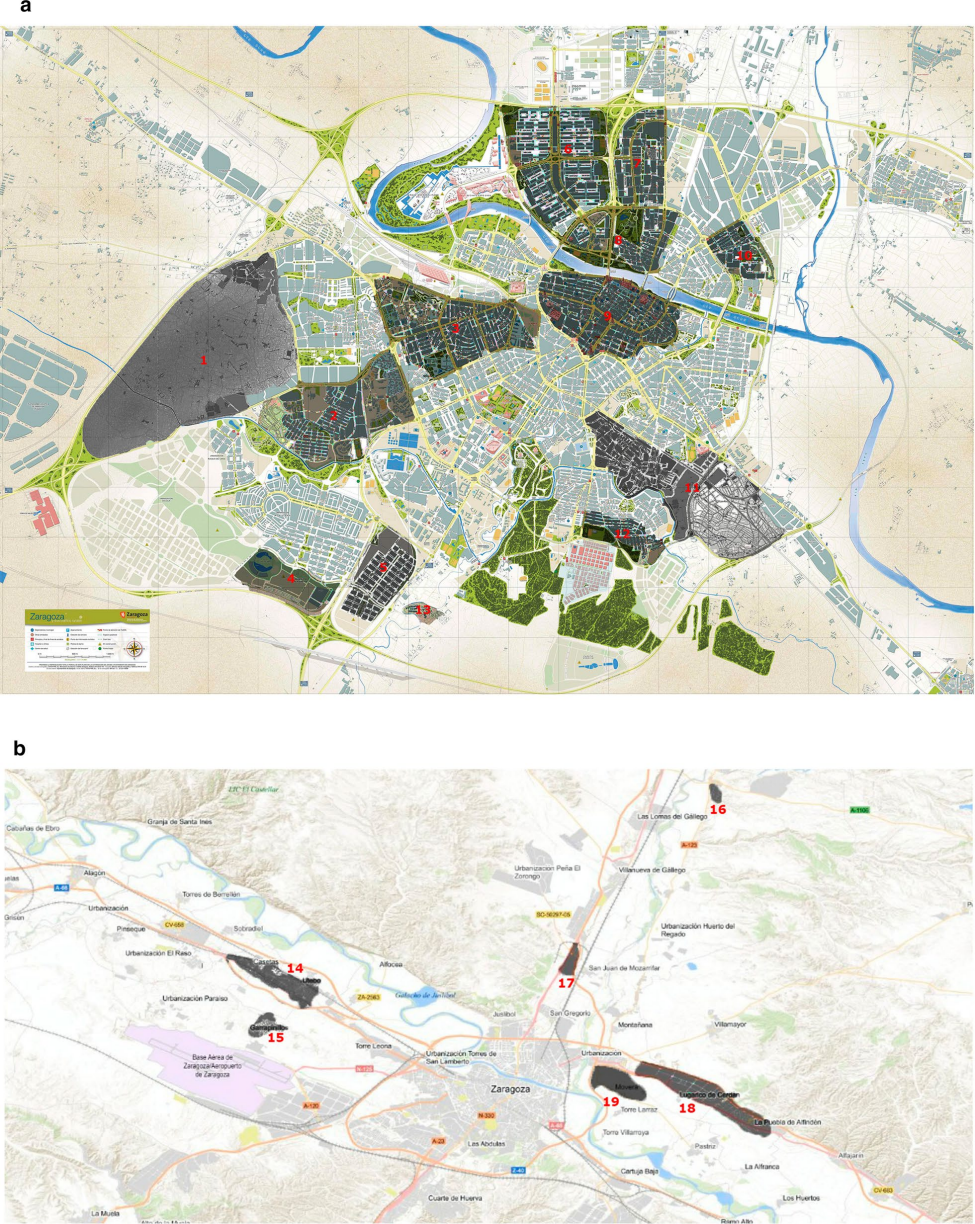
Location of the stray cat colonies in Zaragoza. **a** Urban colonies:* 1* Miralbueno,* 2* Valdefierro,* 3* Delicias,* 4* Valdespartera,* 5* Casablanca,* 6* Actur,* 7* Picarral,* 8* Arrabal,* 9* Centro,* 10* La Jota,* 11* San José,* 12* Torrero,* 13* Urbanización de la Junquera. **b** Peri-urban colonies:* 14* Casetas,* 15* Garrapinillos,* 16* Peñaflor,* 17* San Juan de Mozarrifar,* 18* Malpica,* 19* Movera

The study was carried out in the city of Zaragoza (41°38′58.8948ʺN, 0°53′15.7632ʺW, the Aragon region of Spain) on cats who had lived through at least one transmission season. Based on an expected seroprevalence of 8.5% (the canine seroprevalence in Zaragoza), an accepted 5% deviation from the true prevalence and a confidence level of 95%, the sample size necessary to estimate the seroprevalence was calculated to be 120 cats. The study population comprised 180 stray cats captured in urban and peri-urban areas of Zaragoza (Fig. 1) within the framework of a trap, neuter and release sterilization program run locally to control stray populations.

Captured stray cats were anesthetized with a combination of dexmedetomidine (Dexdomitor®; 15 µg/kg, subcutaneous injection), ketamine (Anaestamine®; 5 mg/kg, subcutaneous injection) and methadone (Semfortan®; 0.3 mg/kg, subcutaneous injection). Data on the breed, age, gender and colony of origin of each cat were recorded. A complete physical examination was carried out before sampling.

Prior to collecting blood, the fur of the cats was trimmed around the jugular region. Sampling consisted of collecting 2 ml of blood aseptically by jugular venepuncture, with the collected volume divided equally between a sterile blood collection tube (to obtain the serum) and a second tube containing ethylenediaminetetraacetic acid (EDTA) anticoagulant (for PCR analysis). Blood and separated serum were stored at − 20 °C until processing. Routine laboratory tests, such as a complete blood count and biochemistry profile, were not performed.

### Diagnostic serological tests

Detection of specific antibodies was performed using three in-house serological techniques: the IFAT, ELISA and WB.

#### Detection of *L. infantum* antibodies by IFAT

The IFAT was performed according to the standard procedures of the World Organization for Animal Health [19], using promastigote forms of the strain MHOM/MON-1/LEM 75 zymodeme MON-1 as a whole-parasite antigen fixed on multi-spot slides (Thermo Fisher Scientific, Waltham, MA, USA). Sera from the captured cats were assayed in serial twofold dilutions from 1:20 to 1:2560. Briefly, a twofold dilution of each serum was applied per well. The slides were incubated for 30 min at 37 °C in a moist chamber, and then washed twice with phosphate buffered saline (PBS) for 5 min and once more with distilled water. After the washing procedure, 20 µl of goat anti-cat IgG-fluorescein isothiocyanate conjugate (Sigma-Aldrich, Saint Louis, MO, USA) diluted 1:64 in 0.2% Evans blue was added to each well. The slides were incubated in a moist chamber at 37 °C for another 30 min in complete darkness and washed again as described above.

After the second washing procedure, a few drops of mounting medium were placed on the cover slips. The slides were examined under a fluorescence microscope (Leica DM750 RH; Leica Microsystems, Wetzlar, Germany) at 400× magnification, and each well was compared to the fluorescence pattern seen in the positive and negative controls. Positive and negative controls were included on each slide. A positive control serum was obtained from a cat from Spain diagnosed with FeL, confirmed by a positive *L*. *infantum* isolation using a biphasic Novy, McNeal and Nicolle blood agar (NNN) medium, and a negative control serum was obtained from a healthy, non-infected indoor cat. The cut-off value for positive sera was 1:80, in accordance with the literature [8]. Two trained researchers examined every IFAT sample, and a third investigator participated if discrepancies arose between results.

#### Detection of *L. infantum* antibodies by a quantitative ELISA

The ELISA was performed on all sera as described previously, with some modifications [20]. Briefly, each plate was coated with 20 µg/ml of crude antigen obtained from *L. infantum* promastigote forms (MHOM/MON-1/LEM 75) in 0.1 M carbonate/bicarbonate buffer (pH 9.6), and incubated overnight at 4 °C. A 100-µl aliquot of cat sera, diluted 1:200 in PBS containing 0.05% Tween 20 (PBST) and 1% dry skimmed milk (PBST-M), was added to each well. The plates were then incubated for 1 h at 37 °C in a moist chamber, following which they were washed and 100 µL of Protein A conjugated to horseradish peroxidase (Thermo Fisher Scientific) diluted 1:20,000 in PBST-M was added. The plates were incubated for 1 h at 37 °C in the moist chamber and were washed again with PBST and PBS as described above. The substrate solution (ortho-phenylene-diamine) and stable peroxide substrate buffer (Thermo Fisher Scientific) were added to each well and the reaction was allowed to develop for 20 ± 5 min at room temperature in the dark. The reaction was stopped by adding 2.5 M H_2_SO_4_ to each well. Absorbance values were read at 492 nm in an automatic microELISA reader (ELISA Reader Labsystems Multiskan, Midland, ON, Canada).

As a positive control (calibrator), each plate included serum from a cat from Spain diagnosed with FeL, confirmed by a positive *L. infantum* isolation using an NNN medium, and as a negative control, serum from a healthy, non-infected cat. The same calibrator serum was used for all assays and plates, with a constant inter-assay variation of < 10%. Plates with an inter-assay variation of > 10% were discarded. All samples and controls were run in duplicate. The results were quantified as ELISA units (EU) compared to the positive control serum used as a calibrator and arbitrarily set at 100 EU. The cut-off was established at 13 EU (mean + 4 standard deviations [SD] of values from 50 indoor cats from northern Spain) and the results above this value were considered to be positive.

#### Detection of *L. infantum* antibodies by WB

Anti-*Leishmania* antibodies were detected by WB using a whole antigen of *L. infantum* promastigotes (MHOM/FR/78/LEM75 zymodeme MON-1), as described by Riera et al. [20] with some modifications. Antigen electrophoresis in 1% sodium dodecyl sulfate/15% polyacrylamide gels together with molecular mass protein standards (Standard Low Range; Bio-Rad, Hercules, CA, USA) was performed on a Mini-Gel AE 6400 Dual Mini Slab Kit (ATTO Corp., Tokyo, Japan). The gels were run at 100 V for 1 h at room temperature.

 Polypeptides were transblotted onto nitrocellulose sheets (0.45-mm pore size, HAWP 304 FO; Millipore Corp., Bedford, MA, USA), which were blocked with 20 mM Tris, 0.13 mM NaCl, pH 7.6 (TS) and 5% skimmed milk, overnight at 4 °C. The sheets were washed in TS and introduced into a multiscreen apparatus (Mini Protean II, Multiscreen Apparatus; Bio-Rad). Sera were diluted 1:200 in TS/1% skimmed milk and 0.2% Tween 20. Then 500 µl of each sample was introduced into each channel of the multiscreen apparatus and incubated for 2 h at 37 °C. Bound immunoglobulins were developed by incubation with a 1:1000 dilution of Protein A peroxidase conjugate (Thermo Fisher Scientific) for 1 h. After the sheets were washed three times with TST and a final time with TS, color was developed with 4-chloro-1-naphthol (Thermo Fisher Scientific) and H_2_O_2_, and the reaction was stopped with tap water after 30 min. The sera were considered to be positive when immunoreactivity from low-molecular-weight polypeptide fractions of 14, 16, 18, 20, 24, 36 and 46 kDa from the *Leishmania* antigen was observed, as previously reported [21, 22].

#### Detection of *L. infantum* DNA by qPCR

A qPCR was used in this study. DNA was extracted from 200 µl of blood by the isolation of nucleic acids according to the protocol of the Quick-DNA Miniprep Plus Kit (Zymo Research, Irvine, CA. USA) and eluted in 50 µl of elution buffer, following the manufacturer’s instructions.

*Leishmania* spp. DNA was detected and quantified by amplification of a kinetoplast minicircle DNA sequence by qPCR [23]. Each amplification was performed in triplicate in a 10-µl reaction mixture containing 1× iTaq supermix with Rox (Bio-Rad), 15 pmol of direct primer (5′-CTTTTCTGGTCCTCCGGGTAGG-3′), 15 pmol of reverse primer (5′-CCACCCGGCCCTATTTTACACCAA-3′), 50 pmol of the labeled TaqMan probe (FAM-TTTTCGCAGAACGCCCCTACCCGCTAMRA) and 2.5 µl of sample DNA. Cycling was performed using the ABI Prism 7900 system (Applied Biosystems [Thermo Fisher Scientific], Foster City, CA, USA) att 94 °C/55 °C for 40 cycles. A non-template control was used in each run as the qPCR negative control. A tenfold dilution series of DNA from promastigotes (MHOM/ES/04/BCN-61, *L. infantum* zymodema MON-1) was used for calibration (serial dilution from 10^5^ to 10^−3^ parasites/ml), allowing the plotting of a standard curve. The qPCR was considered to be positive for *Leishmania* when the quantification cycle (Cq) was < 40 and the amplification was detected in all the replicates.

#### Detection of feline leukemia virus antigens and feline immunodeficiency virus antibodies by an immunochromatographic rapid test

The rapid test (Uranotest FeLV-FIV; URANOVET, Barcelona, Spain) was performed following the instructions of the manufacturer. All tests were stored at room temperature and were performed as described in the instructions supplied with the test kit.

### Statistical analysis

The SPSS software package (SPSS Inc. IBM Corp., Chicago, IL, USA) was used for statistical analysis. Associations between *L. infantum* and the recorded variables (gender, location, colony of origin, type of environment [including urban or peri-urban area] and feline immunodeficiency virus/feline leukemia virus [FIV/FeLV] status) were analyzed. The significance of these differences was assessed using Fisher’s exact test and a Chi-square test. A *P *value < 0.05 was considered to be significant. Agreements between diagnostic techniques were evaluated using Cohen’s kappa statistic (*κ*) as follows: no agreement (*κ* < 0), slight agreement (0 < *κ* < 0.2), fair agreement (0.2 < *κ* < 0.4), moderate agreement (0.4 < *κ* < 0.6), substantial agreement (0.6 < *κ* < 0.8) and almost perfect agreement (*κ* > 0.8).

## Results

### Animals studied

All of the tested cats (97 females, 83 males) were shorthaired breeds, adults (> 1 year old) and assessed as apparently healthy, with no evident systemic or dermatological signs found during the general physical examination. Eighty-two cats came from urban colonies (*n* = 10) and 98 from peri-urban colonies (*n* = 9). Samples of serum for *Leishmania* serological testing and blood for *Leishmania* qPCR were obtained from nearly all of the cats (178/180). A serum sample was not available from one of the cats and blood containing EDTA anticoagulant was not obtained from another. One serum sample was of insufficient volume to perform a serological analysis to detect the presence of FeLV antigens and FIV antibodies but was used for a *Leishmania* serology test.

### Serology and qPCR for *L. infantum*

Among the 179 cats for which samples were available for testing, four were seropositive for *L. infantum* by IFAT, with antibody titers ranging from 1:80 (1 cat) to 1:160 (2 cats) and 1:640 (1 cat). Of these four cats, two, with antibody titers of 1:160 and 1:640, respectively, tested positive for *L. infantum* by all of the diagnostic methods employed; the other two cats tested positive only by ELISA and WB (Table 1).

**Table 1:** Summary of positivity based on different diagnostic tests from all cat colonies

Colony	Location	Gender	ELISA (EU)	IFAT	WB (kDa bands)	PCR blood (Cq)
Colony 1 (*n* = 23)	Urban	Male 15/23	0/15	0/15	4/15 Cat 13 (24, 46) Cat 24 (46) Cat 27 (46) Cat 28 (46)	4/14 Cat 13 (Cq 38) Cat 24 (Cq 36) Cat 27 (Cq 38) Cat 28 (Cq 38)
		Female 8/23	0/8	0/8	1/8 Cat 2 (46)	1/8 Cat 2 (Cq 34)
Colony 2 (*n* = 9)	Urban	Male 1/9	0/1	0/1	0/1	0/1
		Female 8/9	0/8	0/8	0/8	0/8
Colony 3 (*n* = 6)	Urban	Male 3/6	0/3	0/3	2/3 Cat 22 (46) Cat 61 (46)	1/3 Cat 22 (Cq 37)
		Female 3/6	0/3	0/3	0/3	0/3
Colony 4 (*n* = 8)	Urban	Male 5/8	0/5	0/5	1/5 Cat 23 (16)	0/5
		Female 3/8	0/3	0/3	0/3	0/3
Colony 5 (*n* = 2)	Urban	Female 2/2	0/2	0/2	0/2	0/2
Colony 6 (*n* = 15)	Urban	Male 9/15	1/9 Cat 177 (14)	1/9 Cat 177 (1:80)	1/9 Cat 177 (20, 46)	0/9
		Female 6/15	0/6	0/6	1/6 Cat 156 (46)	0/6
Colony 7 (*n* = 3)	Urban	Male 1/3	0/1	0/1	0/1	0/1
		Female 2/3	0/2	0/2	0/2	0/2
Colony 8 (*n* = 7)	Urban	Male 2/7	0/2	0/2	1/2 Cat 89 (14, 20)	0/2
		Female 5/7	0/5	0/5	0/5	0/5
Colony 9 (*n* = 5)	Urban	Male 3/5	0/3	0/3	0/3	0/3
		Female 2/5	0/2	0/2	0/2	0/2
Colony 10 (*n* = 4)	Urban	Male 2/4	0/2	0/2	0/2	0/2
		Female 2/4	0/2	0/2	0/2	0/2
Colony 11 (*n* = 24)	Peri-urban	Male 12/24	0/12	0/12	2/12 Cat 34 (46) Cat 48 (16)	1/12 Cat 34 (Cq 31)
		Female 12/24	1/12 Cat 46 (118)	1/12 Cat 46 (1:640)	3/12 Cat 30 (18) Cat 38 (16) Cat 46 (14, 16, 24, 36, 46)	1/12 Cat 46 (Cq 31)
Colony 12 (*n* = 5)	Peri-urban	Male 2/5	0/2	0/2	0/2	0/2
		Female 3/5	0/3	0/3	0/3	0/3
Colony 13 (*n* = 10)	Peri-urban	Male 4/10	0/4	0/4	0/4	0/4
		Female 6/10	0/6	0/6	1/6 Cat 71 (16, 18)	0/6
Colony 14 (*n* = 4)	Peri-urban	Male 3/4	1/3 Cat 178 (24)	1/3 Cat 178 (1:160)	1/3 Cat 178 (14, 16, 36, 46)	0/3
		Female 1/4	0/1	0/1	0/1	0/1
Colony 15 (*n* = 3)	Peri-urban	Male 1/3	0/1	0/1	0/1	0/1
		Female 2/3	0/2	0/2	0/2	0/2
Colony 16 (*n* = 3)	Peri-urban	Female 3/3	0/3	0/3	1/3 Cat 91 (46)	0/3
Colony 17 (*n* = 21)	Peri-urban	Male 9/21	0/9	0/9	0/9	0/9
		Female 12/21	0/11	0/11	0/11	0/12
Colony 18 (*n* = 13)	Peri-urban	Male 7/13	2/7 Cat 95 (17) Cat 109 (26)	1/7 Cat 95 (1:160)	2/7 Cat 95 (14, 16, 36, 46) Cat 176 (16, 46)	2/7 Cat 94 (Cq 38) Cat 95 (Cq 31)
		Female 6/13	0/6	0/6	1/6 Cat 175 (16, 46)	0/6
Colony 19 (*n* = 15)	Peri-urban	Male 4/15	0/4	0/4	1/4 Cat 40 (36)	0/4
		Female 11/15	0/11	0/11	3/11 Cat 39 (16, 18) Cat 76 (46) Cat 84 (14)	0/11

ELISA, Enzyme-linked immunosorbent assay; EU, ELISA units; IFAT, immunofluorescent antibody test; WB, western blot; Cq, quantification cycle

Ten of the 179 cats tested were seroreactive by IFAT, with antibody titers below the IFAT cut-off: 1:20 in five cats and 1:40 in the other five cats. None of these ten cats tested positive by the other serological techniques or qPCR (Table 1).

Five cats (2.7%) were seropositive to the *L. infantum* antigen (mean ± SD: 39.8 ± 44 EU) by ELISA. One cat (Cat 46) showed a high antibody ELISA titer (118 EU) and tested positive by IFAT, WB and qPCR. The other four ELISA-seropositive cats showed low positivity (range 14–26 EU) (mean ± SD: 20.3 ± 5.7 EU); one of these (Cat 95) was also positive by all four diagnostic methods and a second (Cat 109) was only positive by ELISA. The other 174 cats tested were seronegative by ELISA (mean ± SD: 5.9 ± 1.6 EU). No doubtful results were obtained with this test for any cat (Table 1).

**Fig. 2:**
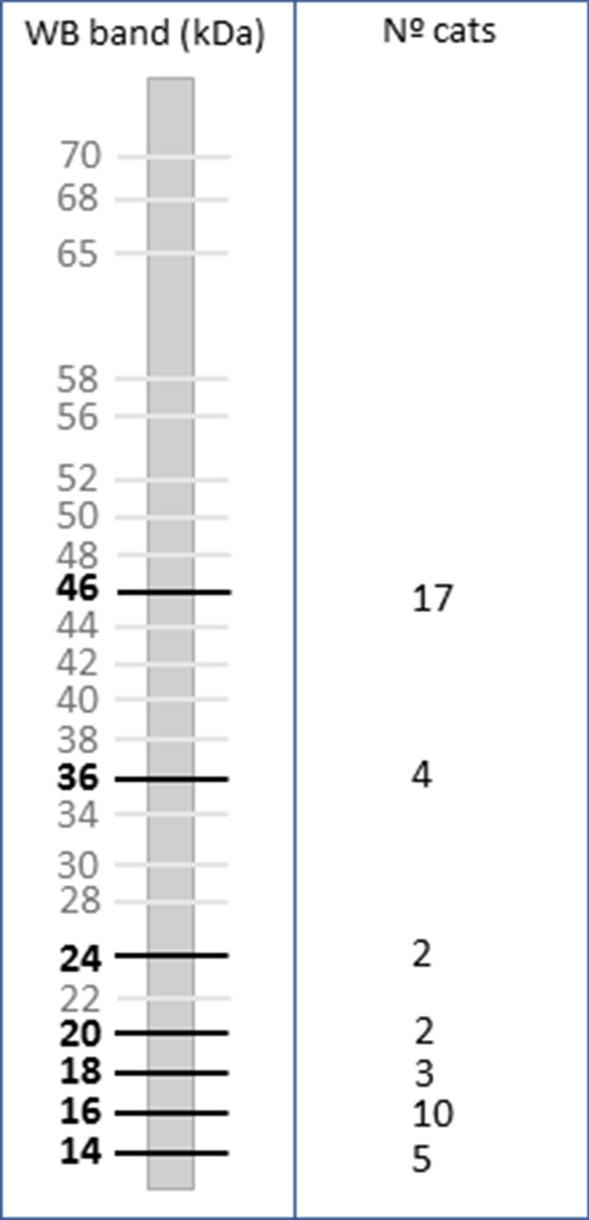
Antibody recognition of *Leishmania infantum* antigens by western blot (*WB*) in 179 cats from Zaragoza (Spain)

In the WB analysis, none of the polypeptide fractions were recognized in 26 cats (14.52%). The sensitivity to the *L. infantum* antigen is reported in Fig. 2. Of these 26 cats, 16 showed only one molecular mass band, seven showed two molecular mass bands, two showed four molecular mass bands and one showed five molecular mass bands.

The most frequent positive band was at 46 kDa (17/26 cats), followed by a band at 16 kDa (10/26). Nine of these 17 cats with a positive band at 46 kDa tested positive for *Leishmania* spp. DNA by qPCR, of which six were seroreactive to the 46-kDa band, one (Cat 13) to the 24- and 46-kDa bands, one (Cat 95) to the 14-, 16-, 36- and 46-kDa bands and one (Cat 46) to 14-, 16-, 24-, 36- and 46-kDa bands (Table 1). Of these 26 cats that tested positive by WB, four also tested positive by IFAT and ELISA and two by qPCR. In total, seven of the 26 cats were qPCR positive.

The molecular analysis detected *Leishmania* spp. DNA in ten of the 179 animals studied (5.6%), two of which also tested positive by all the serological techniques, one tested positive only by qPCR and the rest were also positive by WB (Table 1).

The overall prevalence of *L. infantum* infection was 15.6%, considering a cat to be infected if it tested positive by at least one of the diagnostic techniques.

### Association of *Leishmania* positivity with colonies

Positive results associated with the colony of origin are listed in Table 1. Cats in 11 of the 19 colonies tested positive for *L. infantum* infection, with the majority of these cats located in three colonies: one urban (21.74% of positive cats [5/23], colony 1) and two peri-urban (20.83% of positive cats [5/24], colony 11; and 30.77% of positive cats [4/13], colony 18). In terms of sex, 18.07% of males (15/83) and 7.21% of females (7/97) tested positive by at least one technique.

Two cats, one male (Cat 95) and one female (Cat 46) from different colonies were positive for *L. infantum* infection by all the diagnostic techniques, although physical examination of these two infected cats did not reveal any abnormalities compatible with FeL. Among the 180 cats included in this analysis, two males (Cat 177 and Cat 178) from different colonies yielded a positive result with all of the serological techniques but were negative by qPCR; physical examination of these cats revealed no abnormality.

No significant association was found between positivity for anti-*Leishmania* antibodies and gender, location or type of environment (Table 2). A significant association was found between gender (male) and positivity detected by qPCR (*P* = 0.026).

**Table 2:** Characteristics of a population of stray cats in Zaragoza examined for *Leishmania infantum* infection

Factor	*P* value^a^					
	ELISA positive	IFAT positive	WB positive	PCR positive	Positive by any serological method	Positive by any method
Gender	0.126	0.246	0.210	0.026	0.232	0.194
Colony of origin	0.368	0.604	0.587	0.187	0.432	0.297
Environment (urban/peri-urban)	0.240	0.398	0.698	0.335	0.357	0.224

^a^Associations with a *P* value of < 0.05 were considered to be statistically significant

### Agreement between serological and molecular findings

The seroprevalence of *L. infantum* infection, based on a positive result in any serological test, was 15.1% (95% confidence interval [CI] 10.5–21.1%). The seroprevalence rate according to the different tests was 14.5% for WB (95% CI 10.1–20.5%), 2.8% for ELISA (95% CI 1–6.6%) and 2.2% for IFAT (95% CI 0.7–5.8%). The molecular prevalence was 5.6% (95% CI 2.9–5.6%). The overall prevalence of *L. infantum* infection, based on a positive result from at least one of the diagnostic techniques, was 15.6% (95% CI 10.9–21.6%). Kappa agreement was almost perfect between IFAT and ELISA results (*κ* = 0.89), and fair between IFAT and qPCR (*κ* = 0.26) and ELISA and qPCR (*κ* = 0.24). Agreement was higher between IFAT and WB (*κ* = 0.40) than between ELISA and WB (*κ* = 0.37), and there was a slight agreement between WB and qPCR (*κ* = 0.19).

### Serological prevalence of FeLV and FIV infections

The seroprevalence of FeLV and FIV infections was 4.49% (8/178 cats) and 6.74% (12/178), respectively. Among the 178 cats, eight cats (5 males and 3 females) were seropositive by the immunochromatographic test for FeLV. The presence of antibodies against FIV was detected in five males and seven females.

No significant association was found between positivity for FeLV infection and positivity for anti-*Leishmania* antibodies, gender, location or type of environment (Table 3). In contrast, a significant association was found between FIV infection and positivity detected by IFAT (*P* = 0.023) and ELISA (*P* = 0.037). No other significant associations were found for the other factors evaluated.

**Table 3:** Characteristics of a population of stray cats in Zaragoza examined for feline leukemia virus antigens and feline immunodeficiency virus infections

Factor	*P* value^a^	
	FeLV positivity	FIV positivity
Gender	0.476	0.773
Colony of origin	0.327	0.431
Environment (urban/peri-urban)	0.727	0.551
ELISA positive (*L. infantum*)	0.999	0.037
IFAT positive (*L. infantum*)	0.999	0.023
WB positive (*L. infantum*)	0.599	0.656
PCR positive (*L. infantum*)	0.999	0.999

FeLV, Feline leukemia virus antigens; FIV, feline immunodeficiency virus

^a^Associations with a *P* value of < 0.05 were to beconsidered statistically significant

### Co-infections

Co-infection with *L. infantum* and FIV was detected in two female cats (Cat 177 and Cat 178), no co-infection with *L. infantum* and FeLV was observed and one female cat was co-infected with both retroviruses.

## Discussion

In Spain, the first *Leishmania* infection in cats was described in 1933 [24], and since then the number of natural cases of feline leishmaniosis has increased not only in Spain but also worldwide [25]. In areas endemic for *L. infantum*, dogs and other domestic animals, such as ferrets [26], have been diagnosed with clinical leishmaniosis, but the epidemiological role of the cat is still not completely or clearly understood. In this context, there is an urgent need for epidemiological surveys to ascertain the contribution of infected cats in areas where cases are detected.

Epidemiological studies of cats performed in different regions of Spain report seroprevalences of *L. infantum* ranging from 1.29 to 60% [27, 28]. Surveys using different serological methods have found that the prevalence of *L. infantum* infection in cats in Spain varies from 0.43 to 26% [27, 29] (Table 4).

**Table 4:** Results of epidemiological surveys of feline leishmaniosis in Spain

Year	Region	Type of animal (domestic, mixed, stray)	Number of cats analyzed	Presence of clinical signs and clinicopathological findings	IFAT	ELISA	WB	DAT	PCR (sample)	Direct detection of *L. infantum*	References
2002	Barcelona and Girona	Domestic	117	N.A	N.A	1.7%	N.A	N.A	N.A	N.A	[53]
2002	Aragón	Domestic	50	Immunological alteration	N.A	N.A	N.A	42.00%	N.A	N.A	[32]
2007	Barcelona, Tarragona and Island of Mallorca	Mixed	445	N.A	N.A	5.30–6.30%	N.A	N.A	N.A	N.A	[34]
2007	Southern Spain	Domestic	183	N.A	28.30–60.00%	N.A	N.A	N.A	25.70% (blood)	1.63%	[28]
2008	Barcelona	Domestic	100	Yes; clinical signs or laboratory findings for positive cats inconsistent with leishmaniosis	N.A	N.A	N.A	N.A	3% (blood)	N.A	[13]
2008	Madrid	Domestic	233	Yes; relative lymphocytosis and an increase in alanine aminotransferase value	1.29–4.29%	N.A	N.A	N.A	0.43% (blood)	N.A	[27]
2011	Island of Mallorca	Stray	86	N.A	N.A	N.A	15.70%	N.A	26.00% (blood)	N.A	[29]
2011	Island of Mallorca and Ibiza	Mixed	105	Yes; cutaneous lesions	N.A	13.20%	N.A	N.A	8.70% (blood)	N.A	[11]
2011	Madrid	Domestic	20	N.A. Some cats co-infected with *Tritrichomas foetus*	15.00%	N.A	N.A	N.A	N.A	N.A	[54]
2012	Madrid	Mixed	680	N.A	3.70%	N.A	N.A	N.A	0.60% (blood)	N.A	[55]
2014	Madrid, Toledo and Guadalajara	Stray	346	Yes, 3.2%	3.20%	N.A	N.A	N.A	0.00% (blood)	N.A	[15]
2021	Zaragoza	Stray	180	No	2.23%	2.79%	14.52%	N.A	5.58% (blood)	N.A	This study

N.A., Not available

A study on canine leishmaniosis in Zaragoza found a seroprevalence of 8.5% by IFAT [30]. In the same area, a total of 130 human cases of leishmaniasis were reported from 2000 to 2019 [31]. Nevertheless, the true prevalence of *L. infantum* infection in cats in the Aragon region is unknown; the only published information available is a congress communication on a study of 50 domestic cats, 42% of which were seropositive by the DAT and exhibited immune dysfunction [32].

Epidemiological surveys reporting the presence of anti-*Leishmania* antibodies in feline sera have used a range of techniques, such as IFAT, ELISA, WB and DAT. In general, blood is less suitable for detecting parasitic DNA than bone marrow or the lymph node, but in an epidemiological setting, the procedure to obtain these alternative samples may be impractical and blood is usually the only type of sample available. It should be mentioned that there is a lack of consensus on the ideal biological sample to use in the molecular diagnosis of FeL [33]. In this study, we applied three different serological techniques and a molecular test to evaluate the global prevalence of the infection.

Among the serological techniques used in this survey, positivity of 14.5% was detected by WB in feline serum samples, which is similar to the positivity reported in a study performed in Mallorca (15.70%) [29]. ELISA was slightly more effective than IFAT in terms of detecting antibodies against *L. infantum*, in contrast with another study [12]. The main differences between these three serological techniques that would explain the results obtained are the type of antigen used and the technical method performed to obtain the results. WB is less frequently used for the serological detection of infection than IFAT or ELISA and requires the use of a specialized laboratory and reagents to distinguish the molecular weight of the *L. infantum* antigens present in the serum sample.

In the WB analysis, certain bands associated with the diagnosis were detected with more frequency, particularly a band with the molecular weight of 46 kDa and bands with low molecular weight, such as 14, 16, 18 and 20 kDa; all of these bands are considered to be specific for detecting infection. In our study, the presence of bands at 14, 16, 18, 20, 24, 36 and 46 kDa suggested *L. infantum* infection. In contrast, other authors report that bands at 18 kDa or at 16, 28, 34, 54 and 69 kDa do not indicate *L. infantum* infection [12, 34]. Bands at 14, 16, 36 and 46 kDa were detected in two cats that tested positive for *L. infantum* infection by the other three tests. In particular, the 46-kDa band was detected in nine cats with a positive qPCR result. In asymptomatic infection in humans and dogs, the presence of the polypeptide fractions of 14 and/or 16 KDa together with other bands of low molecular weight indicates infection or exposure [35–37]. In our study, we observed low-molecular-weight bands in 16 cats, three of which gave a positive qPCR result, and the 46-KDa band was observed in two of these three cats. In six cats showing reactivity only against the 46-kDa polypeptide fraction, *Leishmani*a DNA in blood was detected by qPCR. However, more serological and molecular studies are needed to ascertain the specificity of this band and whether it can be used to detect prior contact with the parasite or asymptomatic infection in cats [21, 22, 38, 39].

The cats studied by both WB and ELISA showed different seroprevalence results according to the methodology used (8.38 and 2.79%, respectively), probably due to the different sensitivity and specificity of the techniques.

Small differences in the detection of anti-*Leishmania* antibodies between the ELISA and IFAT techniques could be due to the type of antigen used and the technical method performed to obtain the results. Another influential factor is the interpretation of the results; in IFAT, the interpretation of results is subjective and dependent on the operator´s experience. In this study, a cut-off dilution of 1:80 was applied in the IFAT technique, following the LeishVet guidelines [8]. However, some cats were seropositive below the IFAT cut-off, yet seronegative by WB. In a recent publication, a cut-off dilution of 1:10 was established for seropositive cats with titers between 1:20 and 1:40 [40]. In light of our results, it seems reasonable to believe that the 1:80 cut-off point is suitable for detecting the presence of anti-*Leishmania* antibodies in cats in endemic areas.

In areas endemic for *Trypanosoma* spp. or other *Leishmania* spp., cross-reactions with *L. infantum* must be taken into account when interpreting serological results, but this is not the case for the geographical area investigated in this study. Cross-reactions can occur in serological tests, especially when a whole-parasite antigen is used; thus, the possibility that new vector-borne pathogens in cats might be able to produce a false positive result for *L. infantum* should not be forgotten. However, in our study, all of the serum samples were collected from cats from the city of Zaragoza [41], a region in Spain where *Trypanosoma* species are not present and new vector-borne parasites have not been reported; moreover, *L. infantum* is the causative parasite of FeL in Europe.

The detection of *L. infantum* DNA by qPCR in different matrices has been evaluated in different studies, including non-invasive procedures to obtain the biological material, such as conjunctival swabs [42], or invasive procedures to obtain blood, lymph node and bone marrow samples [33]. Studies on stray cats in Spain have tested blood by qPCR to detect the parasite and found variable prevalence, depending on the study and the endemic region.

In the present study, qPCR positivity in blood material was slightly higher (5.6%) than in a study performed in Madrid, which involved a higher number of cats and did not detect the parasite DNA in blood samples [15]. The differences between our results and those of other epidemiological studies are likely due to the high rate of infection in cat population. Another possibility might be differences in the performance of the sampling technique between studies. Our findings are in disagreement with the suggestion of a recent study that blood is not a suitable tissue for the molecular diagnosis of *L. infantum* infection in cats [18], as the procedure to obtain blood is easy to perform in epidemiological studies.

In a previous study on of *Leishmania* infection in cats, diagnosis was based on the PCR analysis of blood, skin biopsy, bone marrow and conjunctiva, and positive results were obtained in 13%, 18.2%, 16% and 3.1% of the cats, respectively, the conjunctiva being the least frequently positive matrix [33]. Further research comparing other non-invasive samples (buccal swabs, brush sampling) for molecular diagnosis is needed.

The significant association between gender and *L. infantum* positivity by qPCR (*p* = 0.026) could be related to a risk factor. The higher proportion of positive males than females could be due to differences in behaviour that result in males being more exposed to sand fly bites. In general, male cats occupy a larger area than females and they tend to exist on the periphery of colonies, with large territories that may overlap with several groups of females [43]. One possible explanation for the absence of a significant association between the type of environment and positivity detected by serological techniques and/or qPCR is that *L. infantum* infection is present in both urban and peri-urban cat colonies, and therefore any cat in an urban and peri-urban area endemic for *L. infantum* could be potentially exposed.

FeLV and FIV are immunosuppressive diseases that leave cats susceptible to secondary infections, which may impair their welfare and reduce their lifespan [44]. An association between FIV and *L. infantum* is frequently detected [7], and FeL co-infection with FIV is more prevalent than that with FeLV. In our study, a statistical association was found between FIV and seropositivity detected by IFAT (*P* = 0.023) and ELISA (*P* = 0.037). In contrast, no association was observed between FeLV and *L. infantum* regardless of the technique. These results are in agreement with those reported in the literature [8].

This study, which assessed *L. infantum* infection in 180 asymptomatic stray cats from urban and peri-urban areas in the city of Zaragoza (Aragón, Spain), provides additional confirmation of the value of health surveillance programs for the detection and monitoring of diseases that affect domestic and stray animals. The control and elimination of leishmaniasis requires not only the detection of human and canine infection, but also an effective identification and control of other reservoirs as well as the implementation of vector control. These outdoor animal resevoirs are exposed to vector activity, which could lead to a possible source of infection, as occurred in hares in Fuenlabrada (Madrid, Spain) [45]. One study [46] reported that the density of *Phlebotomus perniciosus*, one of the two vectors of *L. infantum* in Spain, was positively correlated with the presence of cats. The opportunistic feeding behavior of *P. perniciosus*, taking blood meals from a range of animal reservoirs, has been demonstrated in Menorca [47] and other Mediterranean foci [48, 49]. The positive correlation of *P. perniciosus* density with cats is of epidemiological importance, because cats may act as additional reservoirs. None of the animals analyzed here by molecular and serological techniques showed clinical signs compatible with leishmaniosis, which supports the hypothesis that cats are likely reservoirs of *L. infantum* [50]. These findings are similar to results reported in dogs [51] and humans [52], both well known for the occurrence of a chronic subclinical infection. Although the blood of asymptomatic animals is not the most suitable sample to detect leishmaniosis infection, parasitemia was found in the blood of ten cats. Two of the qPCR-positive cats also tested positive in all the serological tests used. Analyzing blood samples for *Leishmania* DNA may help to determine the risk of infection transmission in stray cats.

According to the data provided by the Official College of Veterinarians of Zaragoza, there are 72,811 dogs and 4287 cats registered in the metropolitan area of Zaragoza. The cat population could be significantly higher as their registration is not mandatory in the region of Aragon. There are not any stray dogs, whereas stray cats can be seen around the city. Given the seroprevalence found in this study, the role these animals might play in the leishmaniosis cycle should not be underestimated, especially as the cats live close to humans in an endemic area.

Future studies should focus on determining the urban cycle of *L. infantum*, including the presence of other competent urban reservoirs. The role of the cat as a reservoir should be further explored, as well as the control and prevention of infection transmission in stray cats.

## Conclusions

Our results demonstrate the presence of *L. infantum* infection in apparently healthy stray cats in the city of Zaragoza. Parasite DNA was detected in some feline blood samples. The presence of stray cats in urban areas is a factor in the epidemiology of feline infection, given that the evaluated cats were classified as healthy and asymptomatic.

## Data Availability

The datasets supporting the conclusions of this article are included within the article. All analysed data are available from the corresponding author upon reasonable request.

## Abbreviations

Cq: Quantification cycle
DAT: Direct agglutination test
EDTA: Ethylenediaminetetraacetic acid
ELISA: Enzyme-linked immunosorbent assay
EU: ELISA units
IFAT: Immunofluorescent antibody test
NNN: Novy, McNeal and Nicolle blood agar
qPCR: Quantitative real-time PCR
WB: Western blot
